# Upregulation of the pathogenic transcription factor SPI1/PU.1 in tuberous sclerosis complex and focal cortical dysplasia by oxidative stress

**DOI:** 10.1111/bpa.12949

**Published:** 2021-03-30

**Authors:** Till S. Zimmer, Anatoly Korotkov, Susan Zwakenberg, Floor E. Jansen, Fried J. T. Zwartkruis, Nicholas R. Rensing, Michael Wong, Angelika Mühlebner, Erwin A. van Vliet, Eleonora Aronica, James D. Mills

**Affiliations:** ^1^ Department of (Neuro)Pathology Amsterdam Neuroscience Amsterdam UMC University of Amsterdam Amsterdam the Netherlands; ^2^ Center for Molecular Medicine Molecular Cancer Research University Medical Center Utrecht Utrecht the Netherlands; ^3^ Department of Pediatric Neurology, Brain Center University Medical Center Utrecht Utrecht the Netherlands; ^4^ Department of Neurology Washington University Saint Louis MO USA; ^5^ Department of Pathology University Medical Center Utrecht Utrecht the Netherlands; ^6^ Center for Neuroscience Swammerdam Institute for Life Sciences University of Amsterdam Amsterdam the Netherlands; ^7^ Stichting Epilepsie Instellingen Nederland (SEIN) Heemstede the Netherlands; ^8^ Department of Clinical and Experimental Epilepsy UCL London UK; ^9^ Chalfont Centre for Epilepsy Chalfont St Peter UK

**Keywords:** brain inflammation, epilepsy, focal cortical dysplasia, mTOR, oxidative stress, tuberous sclerosis complex

## Abstract

Tuberous sclerosis complex (TSC) is a congenital disorder characterized by cortical malformations and concomitant epilepsy caused by loss‐of‐function mutations in the mTOR suppressors *TSC1* or *TSC2*. While the underlying molecular changes caused by mTOR activation in TSC have previously been investigated, the drivers of these transcriptional change have not been fully elucidated. A better understanding of the perturbed transcriptional regulation could lead to the identification of novel pathways for therapeutic intervention not only in TSC, but other genetic epilepsies in which mTOR activation plays a key role, such as focal cortical dysplasia 2b (FCD). Here, we analyzed RNA sequencing data from cortical tubers and a tsc2^−/−^ zebrafish. We identified differential expression of the transcription factors (TFs) SPI1/PU.1, IRF8, GBX2, and IKZF1 of which SPI1/PU.1 and IRF8 targets were enriched among the differentially expressed genes. Furthermore, for SPI1/PU.1 these findings were conserved in TSC zebrafish model. Next, we confirmed overexpression of SPI1/PU.1 on the RNA and protein level in a separate cohort of surgically resected TSC tubers and FCD tissue, in fetal TSC tissue, and a *Tsc1* ^GFAP−/−^ mouse model of TSC. Subsequently, we validated the expression of SPI1/PU.1 in dysmorphic cells with mTOR activation in TSC tubers. In fetal TSC, we detected SPI1/PU.1 expression prenatally and elevated RNA Spi1 expression in *Tsc1* ^GFAP−/−^ mice before the development of seizures. Finally, *in vitro*, we identified that in astrocytes and neurons *SPI1* transcription was driven by H_2_O_2_‐induced oxidative stress, independent of mTOR. We identified SPI1/PU.1 as a novel TF involved in the pro‐inflammatory gene expression of malformed cells in TSC and FCD 2b. This transcriptional program is activated in response to oxidative stress and already present prenatally. Importantly, SPI1/PU.1 protein appears to be strictly limited to malformed cells, as we did not find SPI1/PU.1 protein expression in mice nor in our *in vitro* models.

## Graphical abstract

Using RNA sequencing data from surgically resected tubers from tuberous sclerosis complex (TSC) patients and tissue from a tsc2^−/−^ zebrafish knockout model we identified the transcription factor SPI1/PU.1 to be upregulated and its targets to be enriched among the differentially expressed genes. Furthermore, we validated these findings in a separate cohort of TSC as well as in the histopathologically similar focal cortical dysplasia (FCD) type 2b, in fetal TSC tissue and an experimental TSC model on the RNA and protein levels. We identified SPI1/PU.1 as a novel transcription factor involved in the pro‐inflammatory gene expression of malformed cells in TSC and FCD 2b and found in vitro evidence for its regulation by oxidative stress.

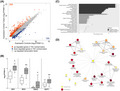

## 1 INTRODUCTION

Tuberous sclerosis complex (TSC) is a genetic disorder that is caused in part by loss‐of‐function mutations in one of two genes, *TSC1* or *TSC2*. The proteins encoded from these genes, tuberin and hamartin, are important regulators of the mammalian target of rapamycin complex 1 (mTORC1) (1). As such, loss‐of‐function mutations in these genes can lead to the hyperactivation of the mTOR pathway resulting in unregulated cell growth and proliferation across numerous organs (1, 2). This multisystem disorder presents with a wide range of clinical manifestations of which the most debilitating affect the central nervous system and include neurodevelopmental delay, autism, and severe seizures (3, 4). Focal cortical malformations, known as cortical tubers, are present in the brains of approximately 90% of patients with TSC and are thought to represent the epileptogenic foci and thus drivers of seizure activity (3, 5). Histological examination of resected cortical tubers reveals a severely distorted cortex, with the apparent loss of cortical layers and heterogeneous entities consisting of a variety of abnormal cell types, including dysmorphic neurons, giant cells, and reactive astrocytes (6). Approximately a third of patients suffer from seizures resistant to currently available anti‐epileptic drugs. The remaining group of patients frequently undergo surgical resection of the cortical tubers. After surgery, 57% of TSC patients achieve seizure freedom and another 18% show a significant reduction in seizure frequency within the first‐year post‐surgery (7, 8). However, for approximately 25% of patients there is no reduction in seizure frequency, and for 3% of these cases there are significant‐associated surgical morbidities (9). With the current treatments targeted at suppressing seizure activity being of mixed effectiveness, there is an urgent need to better understand the cellular and molecular signatures of the epileptogenic brain lesions in TSC so that appropriate treatments can be applied.

At the molecular level, TSC cortical tubers are characterized by an inflammatory response, and activation of the immune system, along with disruption of the extracellular matrix and the blood–brain barrier (10, 11). Furthermore, emerging evidence suggests that oxidative stress (OS) may also play a major role in TSC pathology and the subsequent epileptogenesis (12, 13, 14, 15, 16, 17). The exact roles and important regulatory drivers of the aforementioned processes in the pathological manifestations of TSC have not been fully elucidated. However, current studies point to complex and intricate regulatory networks involving interactions and interplay between microRNAs, loss of heterozygosity, DNA‐methylation changes, and transcriptional regulation (10, 18, 19, 20, 21, 22). Besides the transcription factors (TFs) tumor protein p53 (tp53) and nuclear factor kappa‐light‐chain‐enhancer of activated B cells (NF‐κB), little attention has been given to the transcriptional control of genes associated with TSC (12, 23, 24).

A closely related pathology, which presents as one of the leading causes of refractory childhood epilepsies, is focal cortical dysplasia (FCD) (25). Of all the FCD subtypes, FCD type 2b presents striking histopathological similarities with TSC, such as dysmorphic neurons, dysplastic glia, and balloon cells, which closely resemble giant cells in TSC upon microscopic examination (26). Importantly, FCD 2b is caused by germline or somatic mutations in regulators of mTOR itself, ultimately resulting in mTOR hyperactivity similar to TSC (27). Therefore, FCD 2b and TSC are classified as mTORopathies, characterized by malformed cortical development and frequent epilepsy, likely representing a spectrum of diseases along the mTOR pathway (28). Consequently, TSC could serve as model disease to better understand the pathogenesis of mTORopathies and these insights could also be extrapolated to novel treatments for FCD 2b.

In this study, by leveraging previously published RNA sequencing (RNA Seq) data, we aim to identify TFs that are important regulators of the perturbed gene expression patterns seen in TSC cortical tubers and to investigate if these changes can be extrapolated to FCD 2b. Subsequently, using *in vitro* and *in vivo* models along with resected human brain tissue we delineate the relationship between mTOR activation, the cortical tuber micro‐environment, and the activation of the TFs of interest.

## 2 MATERIALS AND METHODS

### 2.1 Subjects

Surgical and postmortem brain tissues were selected from the archives of the Neuropathology of the Amsterdam University Medical Centres (Amsterdam UMC, location Academic Medical Center (AMC), Amsterdam, the Netherlands), and the University Medical Center Utrecht (UMCU, Utrecht, the Netherlands). Cortical brain samples from patients diagnosed with FCD 2b (RNA n = 8; immunohistochemistry n = 3) or TSC (RNA n = 10; immunohistochemistry n = 3; fetal TSC n = 3) were obtained from brain surgery for intractable epilepsy. Informed consent was acquired for the use of brain tissue for research purposes. Diagnostic workup of the obtained brain specimens was performed independently by two neuropathologists. The diagnosis of FCD 2 was confirmed according to the international consensus classification system proposed for grading FCD 2 (29). All patients with cortical tubers fulfilled the diagnostic criteria for TSC (30), while none of the FCD 2b patients fulfilled the diagnostic criteria for TSC (isolated FCD 2b). Control material (RNA n = 8; immunohistochemistry n = 3) was obtained at autopsy from age‐matched controls, without a history of seizures or other neurological diseases. All autopsies were performed within 24 h after death. Tissue was obtained and used in accordance with the Declaration of Helsinki and the Amsterdam UMC Research Code provided by the Medical Ethics Committee and approved by the science committee of the UMC Utrecht Biobank. Clinical information about the brain samples used in this study is summarized in Tables S1 and S2.

### 2.2 RNA sequencing data

The final differential expression outputs from two different RNA Seq experiments were provided by the data generators on request (10, 31). Dataset 1 was a comparison between TSC cortical tuber (n = 12) and matched controls (n = 10), dataset 2 was a comparison between *tsc2* ^−/−^ zebrafish (n = 4) and wild type (n = 4). For the full details on RNA Seq protocols used and differential expression analysis we refer to the relevant papers.

### 2.3 Transcription factor enrichment analysis

The TFs that were among the genes upregulated in TSC cortical tubers from the aforementioned RNA Seq analysis were selected for further analysis. The TFs tp53 and NF‐κB, although not upregulated at the gene level, were also included as they have previously been associated with TSC. First, all target genes for each TF were extracted from the ChIP‐X database (32). The ChIP‐X database consists of gene–TF relationships inferred from ChIP‐chip, ChIP‐Seq, ChIP‐PET, and DamID experiments. Of the NF‐κB family members, RELB proto‐oncogene (*RELB*), RELA proto‐oncogene (*RELA*), nuclear factor kappa B subunit 1 (*NFKB1*), nuclear factor kappa B subunit 2 (*NFKB2*), and REL proto‐oncogene (REL), only *RELA* appeared in the ChIP‐X database so it was used to assess NF‐κB‐binding potential. Next, the upregulated genes were assessed for over‐representation of target genes for each TF using hypergeometric testing. The gene list was considered enriched for TF targets when the *p*‐value was <0.05.

To assess the upregulated genes in the *tsc2* ^−/−^ zebrafish for enrichment of gene targets for the same set of TFs, a similar approach was employed. First, the target genes listed for each TF was converted to zebrafish homologs using BioMart (33, 34). Using the converted gene targets the upregulated genes were assessed for over‐representation of target genes for each TF using hypergeometric testing. The gene list was considered enriched for TF targets when the *p*‐value was <0.05.

### 2.4 Pathway and gene ontology enrichment analysis

The genes that were upregulated when TSC cortical tubers were compared to control tissue were passed to the R package ReactomePA for pathway enrichment analysis (35). The ReactomePA package allows for enrichment analysis using the reactome pathway database (36). Those pathways with a Benjamini–Hochberg adjusted *p*‐value < 0.05 were considered significant.

The upregulated genes that were targets of each TF of interest were passed to the R package clusterProfiler for a gene ontology (GO) over‐representation enrichment analysis (37). The genes were assessed for enrichment at the GO levels of “cellular compartment,” “biological process,” and “molecular function.” Those GO with a Benjamini–Hochberg adjusted *p*‐value <0.05 were considered significant.

### 2.5 *Tsc1*^GFAP−/−^ mice

All animal experiments in this study were approved by the Washington University Animal Welfare committee. *Tsc1* ^flox/flox^‐GFAP‐Cre knockout (*Tsc1* ^GFAP−/−^) mice with conditional inactivation of the *Tsc1* gene in astrocytes and neurons were generated as described previously (38). *Tsc1* ^flox/+^‐GFAP‐Cre and *Tsc1* ^flox/flox^ littermates have previously been found to have no abnormal phenotype and were used as controls in these experiments. Whole cortex and hippocampus were collected from 2‐week‐old and 2‐month‐old animals (n = 5 animals per group). While 2‐week‐old *Tsc1* ^GFAP−/−^ mice do not have any neurological abnormalities and seizures, 2‐month‐old animals typically present with chronic recurrent seizures (39). For quantitative real‐time PCR, tissues were mechanically minced and homogenized in Qiazol Lysis Reagent (Qiagen Benelux, Venlo, the Netherlands). Subsequent RNA isolation was done using the miRNeasy Mini kit (Qiagen Benelux, Venlo, the Netherlands) according to the manufacturer's instructions.

### 2.6 Immunohistochemistry on human and mouse brain tissue

Human brain tissue was either snap frozen in liquid nitrogen and stored at −80°C for RNA analysis or fixed in 10% neutral buffered formalin and embedded in paraffin for immunohistochemistry. Paraffin‐embedded tissue was sectioned at 5 µm, mounted on pre‐coated glass slides (Star Frost, Waldemar Knittel Glasbearbeitungs, Braunschweig, Germany) and used for immunohistochemical stainings. Sections were deparaffinated in xylene, and ethanol (100%, 96%) and incubated for 20 min in 0.3% H_2_O_2_ diluted in methanol, to block any residual endogenous peroxidase activity. Antigen retrieval was performed using a pressure cooker in 0.01 M sodium citrate buffer (pH 6.0) at 121°C for 10 min. This was followed by washes in phosphate‐buffered saline (PBS; 0.1 M, pH 7.4) and incubation with primary antibody against SPI1 (SPI1/PU.1; human tissue: rabbit monoclonal, clone EP18, Bio SB, Santa Barbara, CA, USA; 1:100; rabbit polyclonal, Thermo Fisher Scientific, Waltham, MA, USA; 1:200; mouse tissue: rabbit monoclonal, clone 9G7, Cell Signaling Technologies, Danvers, MA, USA; 1:100) or immune regulatory factor 8 (IRF8; mouse monoclonal, clone E9, Santa Cruz, Dallas, TX, USA; 1:50) in antibody diluent (VWR International, Radnor, PA, USA) at 4°C overnight. The next day, sections were washed in PBS and then, stained with a polymer‐based peroxidase immunohistochemistry detection kit (Brightvision plus kit, Immunologic, Duiven, the Netherlands) according to the manufacturer's instructions. Staining was performed using Bright 3,3'‐diaminobenzidine (DAB) substrate solution (Immunologic, Duiven, the Netherlands). The reaction was stopped by washing twice in distilled water. Finally, sections were counterstained with Hematoxylin Mayer's solution (Klinipath, Breda, the Netherlands), dehydrated in alcohol and xylene, and coverslipped using Pertex (VWR International, Radnor, PA, USA).

Double labeling of SPI/PU.1 was performed with neuronal nuclear protein (NeuN; mouse monoclonal, clone MAB377; Merck‐Millipore, Temecula, CA, USA; 1:2000), histocompatibility complex II (HLA‐DR; mouse monoclonal, Agilent, Santa Clara, CA, USA; 1:100), ionized calcium‐binding adapter molecule 1 (Iba‐1; rabbit polyclonal, WAKO, Osaka, Japan, 1:2000), glial fibrillary acidic protein (GFAP; mouse monoclonal, clone GA5, Sigma‐Aldrich, St. Louis, MO, USA; 1:4000), phosphorylated S6 ribosomal protein (pS6; rabbit monoclonal, clone 91B2, Cell Signaling Technologies, Danvers, MA, USA; 1:100), or nitric oxide synthase 2 (iNOS; rabbit polyclonal, Abcam, Cambridge, UK; 1:250). For red and blue IHC, sections were incubated at 4°C overnight with SPI1 primary antibody. The next day, sections were washed with PBS and incubated with Brightvision poly‐alkaline phosphatase (AP) anti‐rabbit (Immunologic, Duiven, the Netherlands) for 30 min at room temperature and again washed with PBS. AP activity was visualized with the AP substrate kit III Vector Blue (SK‐5300, Vector Laboratories Inc., Burlingame, CA, USA). Subsequently, sections were cooked in citrate buffer and then, washed with PBS. Incubation with anti‐pS6 antibody was performed at 4°C overnight in antibody diluent (VWR International, Radnor, PA, USA). Sections were processed with a Brightvision kit as described in the preceding text for 30 min at room temperature. Staining was developed using 3'‐amino 9'‐ethylcarbazole substrate solution (AEC, Sigma‐Aldrich, St. Louis, MO, USA). Sections incubated without the primary antibody were essentially blank.

For immunofluorescent labeling, sections were incubated with the respective primary antibodies overnight in antibody diluent (VWR International, Radnor, PA, USA) at 4°C. The next day, sections were washed with PBS and incubated with Alexa Fluor 568 goat anti‐rabbit and Alexa Fluor 488 donkey anti‐mouse antibody (Invitrogen, Eugene, OR, USA, 1:200) in antibody diluent (VWR International, Radnor, PA, USA) for 2 h at room temperature, mounted with Vectashield (Vector Laboratories Inc., Burlingame, CA, USA) and visualized using Leica Confocal Microscope TCS SP8 X DLS (Leica, Son, the Netherlands) at 200x magnification (bidirectional X, speed 600 Hz, pinhole 1.00 AU).

For Spi1/Pu.1 cell count in *Tsc1* ^GFAP−/−^ mice, two pictures from each hemisphere per animal in the posterior parietal association area were taken. Cells were manually counted and are plotted as total cell count per animal from both hemispheres.

### 2.7 *In situ* hybridization

Paraffin‐embedded brain tissue was deparaffinized and underwent antigen retrieval as described in the previous paragraph. The oligonucleotide probe for *Spi1* (Table S3) contained LNA modification, 2‐o‐methyl modification, and a digoxygenin (DIG) label (Qiagen Benelux, Venlo, the Netherlands). Sections were incubated with 100 nM of the probe in hybridization mix (600 mM NaCl, 10 mM HEPES, 1 mM EDTA, 5x Denhardt's, and 50% Formamide) for 90 min at 56°C. Sections were washed with 2x saline‐sodium citrate (SSC) for 2 min, 0.5x SSC for 2 min, and 0.2x SSC for 1 min. After washing with sterile PBS, sections were blocked for 15 min with 1% bovine serum albumin, 0.02% Tween 20, and 1% normal goat serum (NGS). Hybridization was detected with AP‐labeled anti‐DIG (Roche Applied Science, Basel, Switzerland). Nitro‐blue tetrazolium chloride (NBT)/5‐bromo‐4‐chloro‐3‐indolyl phosphate p‐toluidine salt (BCIP) was used as chromogenic substrate for AP (1:50 diluted in NTM‐T buffer (100 mM Tris, pH 9.5; 100 mM NaCl; 50 mM MgCl_2_; 0.05% Tween 20). For double labeling, slides were developed with Vector blue substrate instead. Thereafter, slides were blocked with 10% NGS in PBS for 30 min at room temperature followed by antibody and AEC development as described. Negative control assays were performed without probes (sections were blank).

### 2.8 RNA isolation & quantitative real‐time PCR

For RNA isolation, human tissues as well as cell culture material were homogenized in 700 µL Qiazol Lysis Reagent (Qiagen Benelux, Venlo, the Netherlands). Total RNA was isolated using the miRNeasy Mini kit (Qiagen Benelux, Venlo, the Netherlands) according to the manufacturer's instructions. The concentration and purity of RNA were determined at 260/280 nm using a Nanodrop spectrophotometer (Thermo Fisher Scientific, Waltham, MA, USA). For quantitative real‐time PCR, 250 ng of cell culture‐derived total RNA or 500 ng tissue‐derived total RNA was reverse‐transcribed into cDNA using oligo‐dT primers. Quantitative PCRs were run on a Roche Lightcycler 480 thermocycler (Roche Applied Science, Basel, Switzerland) using the reference genes chromosome 1 open reading frame 43 (*C1orf43*) and elongation factor 1‐α (*Ef1*‐*α*) for human mRNA and hypoxanthine phosphoribosyltransferase 1 (*Hprt*) and TATA‐Box‐binding protein (*Tbp*) for mouse mRNA (Table S4 for primer sequences). Quantification of data was performed using LinRegPCR as described elsewhere (40).

### 2.9 Cell cultures

Primary fetal astrocyte‐enriched cell cultures were obtained from human fetal brain tissue (cortex, 14–19 gestational weeks) from medically induced abortions. All material was collected from donors from whom written informed consent for the use of the material for research purposes was obtained by the Bloemenhove kliniek (Heemstede, the Netherlands). Tissue was obtained in accordance with the Declaration of Helsinki and the Amsterdam UMC Research Code provided by the Medical Ethics Committee. Cell isolation was performed as described previously (40). Briefly, large blood vessels were removed, after which the tissue was mechanically minced into smaller fragments. Tissue was enzymatically digested by incubating at 37°C for 30 min with 2.5% trypsin (Sigma‐Aldrich, St. Louis, MO, USA). Tissue was washed with incubation medium containing Dulbecco's modified Eagle's medium (DMEM)/HAM F10 (1:1) medium (Thermo Fisher Scientific, Waltham, MA, USA), supplemented with 100 units/ml penicillin, 100 µg/ml streptomycin, 1% glutamine (Thermo Fisher Scientific, Waltham, MA, USA), and 10% fetal calf serum (FCS; Thermo Fisher Scientific, Waltham, MA, USA) and triturated by passing through a 70 µm mesh filter. The cell suspension was incubated at 37°C, 5% CO_2_ for 48 h to let glial cells adhere to the culture flask before it was washed with PBS to remove excess myelin and cell debris.

SH‐SY5Y cells were cultured in DMEM/HAM F‐12 supplemented with 100 units/ml penicillin, 100 µg/ml streptomycin, and 10% FCS. Cultures were subsequently refreshed twice a week. Primary cultures reached confluence after 2–3 weeks.

TSC cultures were derived from surgical brain tissue (cortex) obtained from patients undergoing epilepsy surgery at the Wilhelmina Children's Hospital of the University Medical Center Utrecht (UMCU, Utrecht, the Netherlands). All cases were reviewed and diagnosed as described in the preceding text. Cultures were established and cultured in the same manner as fetal astrocyte‐enriched cultures.

Cell cultures for experiments were obtained by trypsinizing confluent cultures and sub‐plating onto poly‐L‐lysine (PLL, 15 µg/ml, Sigma‐Aldrich, St. Louis, MO, USA)‐precoated 12‐well plates (Greiner Bio‐One, Kremsmünster, Austria); 5x10^4^ cells/well were used for RNA isolation and subsequent PCR. TSC‐derived astrocyte cultures were used at passage 2–5 for all experiments. Human fetal astrocytes were treated with 500 µM of H_2_O_2_ for varying periods (Sigma‐Aldrich, St Louis, MO, USA), 100 nM Rapamycin (VWR International, Radnor, PA, USA) or 10 ng/ml recombinant IL‐1β for 24 h (PeproTech, Rocky Hill, NJ, USA) in complete culture medium at 37°C and 5% CO_2_. SH‐SY5Y cells were treated with indicated concentrations H_2_O_2_ for 3 h in culture medium containing 1% FCS. For Rapamycin treatment, rapamycin was applied 1 hour in advance of the 6‐hour H_2_O_2_ stimulation.

### 2.10 Statistical analysis

Statistical analysis was performed with GraphPad Prism software version 5.01 (GraphPad software Inc., La Jolla, CA, USA) using the nonparametric Mann–Whitney U test or Kruskal–Wallis test followed by Dunn's *post hoc* analysis for multiple groups. *p* < 0.05 was assumed to indicate a significant difference. Correlation analysis was performed using Spearman's rank correlation. Data are displayed as bar graphs with standard deviation (SD) or Tukey's style box plot with interquartile range from 25^th^ to 75^th^ percentile plus outliers.

## 3 RESULTS

### 3.1 Identification of SPI1/PU.1 as a pathogenic regulator of the TSC cortical tuber transcriptional network

**FIGURE 1:**
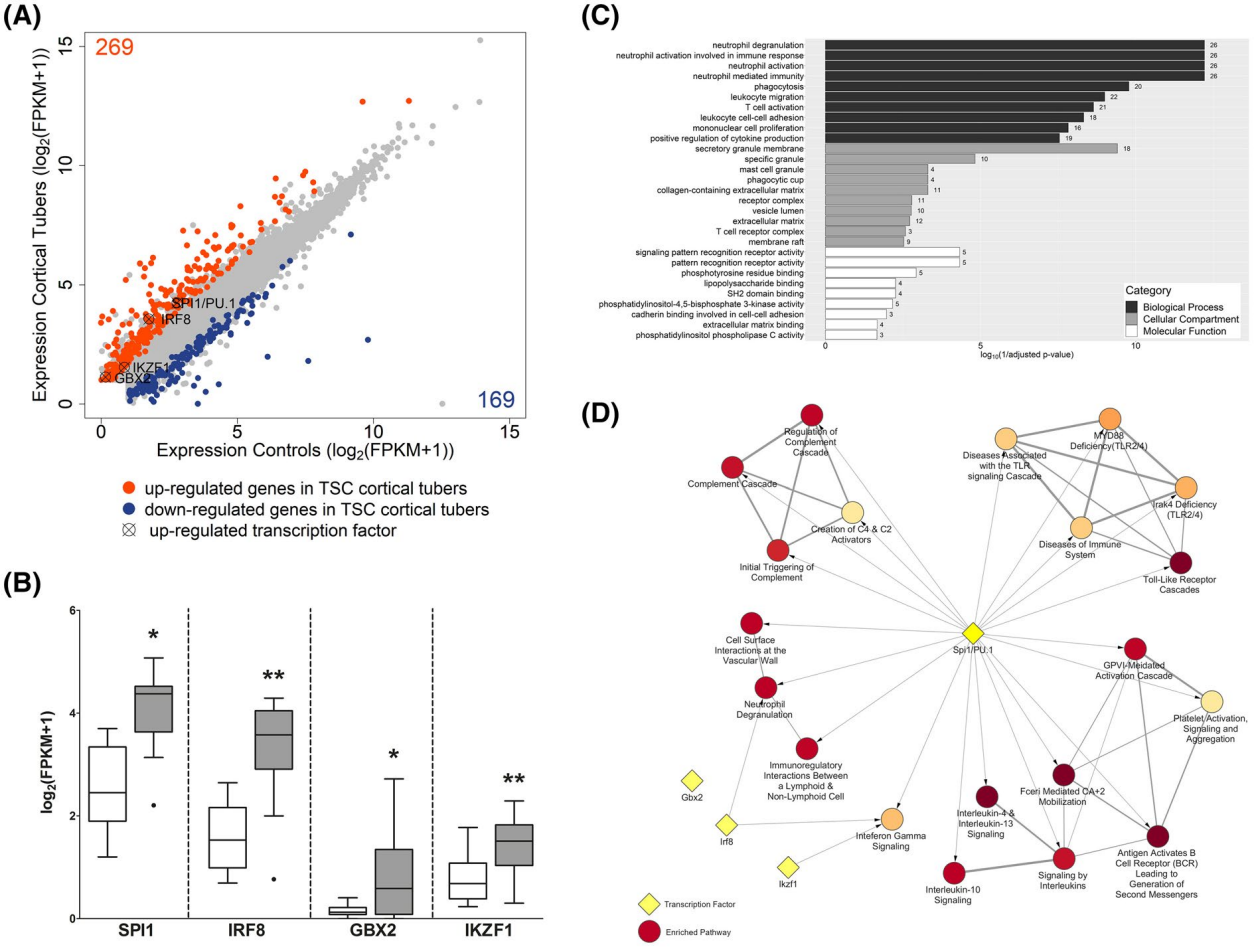
Identification of SPI1, IRF8, GBX2, and IKZF1 as novel transcription factors in TSC cortical tuber. Differential expression analysis of cortical tuber tissue versus age‐matched cortical control samples derived from autopsy revealed 269 upregulated (orange) and 169 downregulated (blue) genes. Four potentially important transcriptional regulators were identified from the upregulated genes (cross circle) (A). Comparison of log_2_FPKM values from cortical tuber tissue versus age‐matched cortical control tissue from autopsy (B). GO enrichment analysis of the top 10 enriched pathways of 139 genes predicted to be targeted by SPI1/PU.1 identified predominantly immune system‐associated GO terms (C). Reactome pathway enrichment analysis of the upregulated genes from RNA Seq revealed SPI1/PU.1 to target multiple genes in the top 20 most enriched pathway. IRF8 targeted 2 pathways, while GBX2 and IKZF1 targeted only 1 pathway. All pathways shown here have an adjusted *p*‐value < 0.007, with the yellow nodes indicating higher *p*‐values and lower *p*‐values indicated by dark red nodes (D). (B) Data presented as Tukey's box plot. RNA Seq n = 10 autopsy control cortex samples versus n = 12 TSC cortical tubers. Statistical test used: modified *t* test

To identify the transcriptional activators that are putative regulators of the TSC cortical tuber transcriptional network, we reanalyzed the RNA Seq data outputs produced by Mills et al. 2017 (10). In this study, 269 genes were identified as upregulated (adjusted *p*‐value < 0.05) and 169 genes were identified as downregulated (adjusted *p*‐value < 0.05) in the TSC cortical tubers when compared to the controls (Figure 1A). The 269 upregulated genes were assessed for the presence of genes associated with the GO term: DNA‐binding transcription factor activity (GO:0003700). Among the 24 upregulated genes that appeared in this GO term five encode for TFs, of which four also appear in the ChIP‐X database (32). Based on this, the TFs SPI1 Proto‐Oncogene (SPI1/PU.1), Interferon Regulatory Factor 8 (Irf8), Gastrulation Brain Homeobox 2 (Gbx2), and IKAROS Family Zinc Finger 1 (Ikzf1) were selected for further analysis (Figure 1A). Of these TFs, at the gene level, *SPI1* had the highest expression in TSC cortical tubers (17.91 fragments per kilobase million (fpkm)), followed by *IRF8* (10.78 fpkm), while *GBX2* (1.18 fpkm) and *IKZF1* (1.89 fpkm) were both expressed at low levels (Figure 1B).

Next, the genes upregulated in the TSC cortical tubers were assessed for enrichment of target genes for each of the four selected TFs. Based on the ChIP‐X database, of the 269 upregulated genes SPI1/PU.1 was a putative transcriptional activator of 136, Irf8 targeted 24, and Ikzf1 and Gbx2 were predicted to be involved in the transcriptional regulation of 4 and 2 genes, respectively (Table 1). Interestingly, 18 of the 24 gene targets of Irf8 were also predicted targets of SPI1/PU.1. Subsequently, hypergeometric testing was performed to assess if there was a significant enrichment of gene targets for each TF; the upregulated genes were significantly enriched for SPI1/PU.1 (*p*‐value = 2.10 × 10^−7^) and Irf8 (*p*‐value = 0.0009) gene targets, but not for Gbx2 (*p*‐value = 0.56) or Ikzf1 (*p*‐value = 0.08) targets. By comparison the genes that form the NF‐κB protein complex, RELB proto‐oncogene (*RELB*), RELA proto‐oncogene (*RELA*), nuclear factor kappa B subunit 1 (*NFKB1*), nuclear factor kappa B subunit 2 (*NFKB2*), and REL proto‐oncogene (REL) along with *TP53* were not differentially expressed, and the TFs themselves were predicted to target 73 (*p*‐value = 1.23 × 10^−7^) and 36 genes of the differentially expressed gene set (*p*‐value = 0.06), respectively (Figure S1A, Table 1).

**TABLE 1:** List of identified transcription factors from human and zebrafish RNA sequencing

Human (Control Tissue vs. TSC cortical Tubers)							Zebrafish (WT v TSC2^−/−^)					
Transcription factor	Description	log2(FC)	Adjusted *p*‐value	Target genes (expressed)	Target genes (upregulated)	Enrichment *p*‐value	Ortholog	log2(FC)	Adjusted *p*‐value	Target genes (expressed)	Target genes (upregulated)	Enrichment *p*‐value
Spi1/PU.1	SPI1 proto‐oncogene	1.607	0.016	6020	136	2.10E‐07	spi1b	1.431	0.041	6170	737	4.31E‐23
Irf8	Interferon regulatory factor 8	2.184	0.004	754	24	0.0009	irf8	0.572	0.224	720	78	0.039
Gbx2	Gastrulation brain homeobox 2	3.360	0.039	185	2	0.56	gbx2	0.017	0.986	197	6	0.999
Ikzf1	Ikaros family zinc finger	1.230	0.004	101	4	0.08	ikzf1	0.962	0.039	143	16	0.202
Tp53	Tumor protein p53	0.268	0.967	3930	73	0.068	tp53	0.572	0.010	4362	353	0.999
Rel	REL Proto‐Oncogene, NF‐KB Subunit	−0.296	0.925	NA	NA	NA	rel	−0.682	0.020	NA	NA	NA
Rela	RELA Proto‐Oncogene, NF‐KB Subunit	0.074	0.998	870	36	1.24E‐07	rela	−0.694	0.431	967	117	0.014
Relb	RELB Proto‐Oncogene, NF‐KB Subunit	0.720	0.511	NA	NA	NA	relb	0.663	0.049	NA	NA	NA
Nfkb1	Nuclear Factor Kappa B Subunit 1	0.340	0.860	NA	NA	NA	nfkb1	0.032	0.983	NA	NA	NA
Nfkb2	Nuclear Factor Kappa B Subunit 2	0.508	0.752	NA	NA	NA	nfkb2	0.288	0.332	NA	NA	NA

Subsequently, the expression of *SPI1* was correlated with the expression of its putative target genes. Of the 136 target genes, SPI1/PU.1 was highly correlated (ρ > 0.70, *p*‐value < 0.05) with 99 including complement C1q B chain (*C1QB*) (*ρ* = 0.948), complement C3 (*C3*) (*ρ* = 0.908), triggering receptor expressed on myeloid cells 2 (*TREM2*) (*ρ* = 0.879), interleukin 1 beta (*IL1B*) (*ρ* = 0.817), and *IRF8 (ρ* = 0.87), (Table S1). To assess the function of the 136 putative target genes, a GO enrichment analysis was performed at the level of cellular compartment, biological process, and molecular function (Table S2). The top enriched GO terms for each level included terms related to the extracellular matrix, immune processes, inflammatory terms, alterations to receptor activity and binding (Figure 1C).

Next, a reactome pathway enrichment analysis of the upregulated genes was performed. In total 39 pathways were identified as enriched (adjusted *p*‐value < 0.05) the majority of which were related to inflammation and immune responses (Table S3). To evaluate which of the enriched reactome pathways may be under the transcriptional control of one of the TFs of interest, the number of gene targets of each TF in each pathway were assessed. All top 20 enriched pathways had multiple (number of genes > 1) SPI1/PU.1 gene targets, while Irf8, Ikzf1, and Gbx2 targeted 2, 1, and 0 pathways, respectively (Figure 1D). In comparison, tp53 had multiple gene targets in 19 pathways and NF‐κB had multiple gene targets in 14 pathways (Table S3).

Finally, by leveraging a RNA Seq analysis of a TSC zebrafish model by Scheldeman et al. 2017, we assessed the conservation of the TFs of interest across an *in vivo* model of TSC (31). Of the zebrafish homologs of the four TFs of interest, the *SPI1* homolog *spi1b* (adjusted *p*‐value = 0.041) (Figure S1B) along with the *IKZF1* homolog *ikzf1* (adjusted *p*‐value = 0.039) were upregulated in the TSC zebrafish model. The expression of the zebrafish homologs of *GBX2* and *IRF8* did not differ (Figure S1B). After converting the target genes extracted from the ChIP‐X database to their zebrafish homologs, the upregulated genes in the TSC zebrafish model were assessed for enrichment of TF targets. Of the 1820 genes upregulated in the TSC zebrafish model, spi1b targeted 737 genes, irf8 targeted 78 genes, while ikzf1 and gbx2 targeted 16 and 6 genes, respectively (Table 1). Hypergeometric testing revealed an enrichment for spi1b (*p*‐value = 4.3x10^−23^) and irf8 gene targets (*p*‐value = 0.0388), while there was no enrichment for target genes of gbx2 and ikzf1. Again, the TFs tp53 and NF‐κB were assessed at the gene expression level in the TSC zebrafish model, *tp53* was upregulated (adjusted *p*‐value = 0.01) along with the NF‐κB family member *rel* (adjusted *p*‐value = 0.0486). However, the expression of the members *rela* (adjusted *p*‐value = 0.43), *nfkb1* (adjusted *p*‐value = 0.98), and *nfkb2* (adjusted *p*‐value = 0.33) did not differ, while the expression of family member *relb* was lower (adjusted *p*‐value = 0.02) (Figure S1C). In terms of transcriptional control, tp53 was predicted to target 353 (*p*‐value = 0.999) of the upregulated genes and NF‐κB was predicted to target 117 (*p*‐value = 0.014) of the upregulated genes (Table 1).

Based on the upregulation of *SPI1* in the RNA Seq data, enrichment of correlated gene targets among the upregulated genes, the predicted involvement in pathways relevant to TSC molecular pathogenesis, along with the conservation of expression patterns and enrichment of target genes in the TSC zebrafish model, we decided to focus predominately on the TF SPI1/PU.1.

### 3.2 SPI1/PU.1 is localized in malformed cells in TSC cortical tubers and FCD 2b foci

**FIGURE 2:**
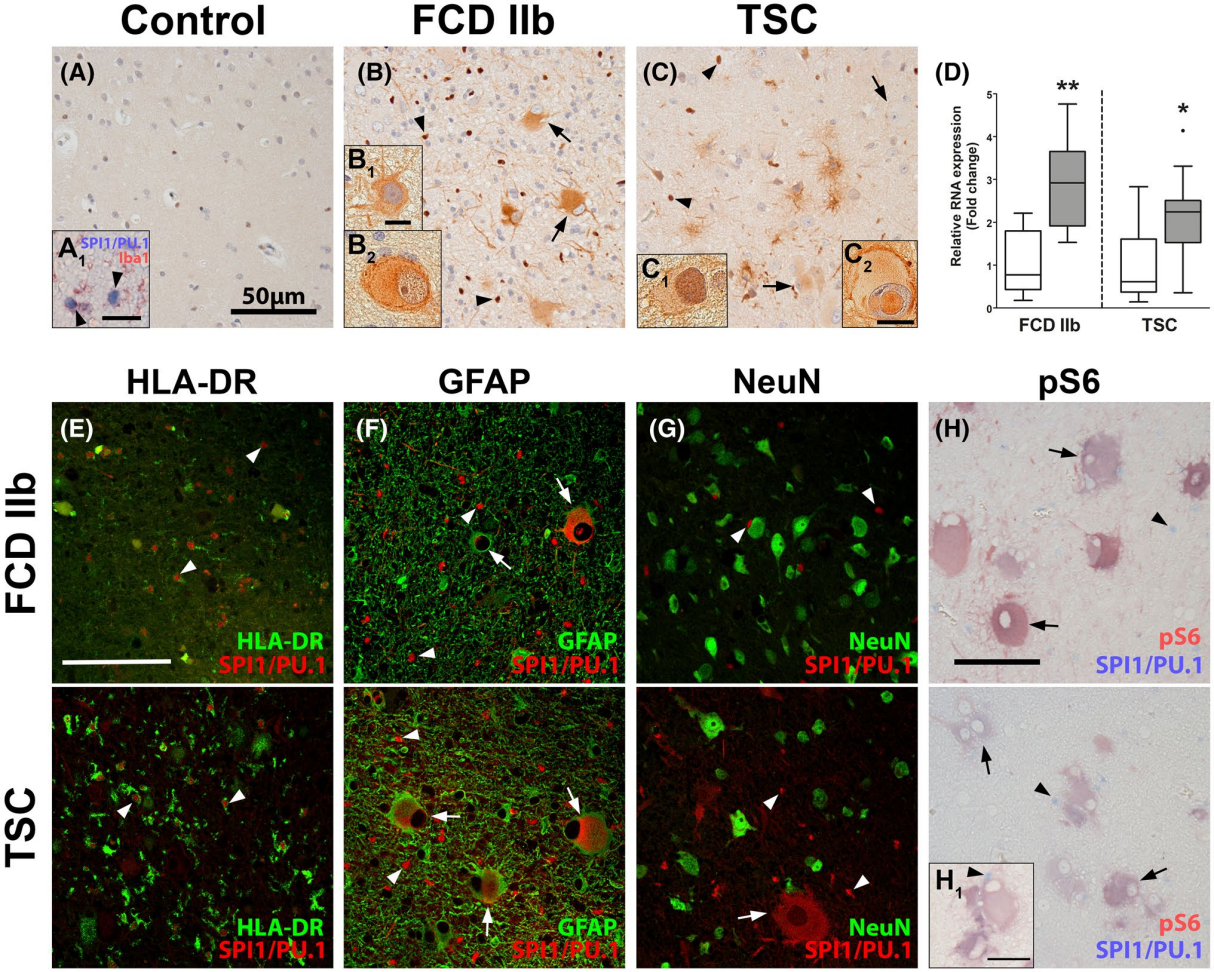
SPI1/PU.1 is highly expressed in malformed cells in TSC and FCD 2b lesions. Autopsy control cortex showed SPI1/PU.1 expression solely in microglia (A, A_1_). In TSC and FCD 2b expression of SPI1/PU.1 was found in microglia (arrowheads) but also malformed cells and dysmorphic neurons (arrows) (B, B_1_, C). Moreover, SPI1/PU.1 expression could also be found in the nuclei of malformed cells (B_2_, C_1_, C_2_). Analysis of RNA expression in tissue from resected TSC tuber and FCD 2b lesion of an independent cohort showed higher expression of *SPI1* compared to control (D). Co‐labeling with cell markers HLA‐DR, GFAP, and NeuN revealed SPI1/PU.1 expression in microglial nuclei (arrowheads) and GFAP^+^NeuN^−^ malformed cells (arrows) in FCD 2b and TSC tissue (E–G). Moreover, malformed cells displayed nuclear SPI1/PU.1 expression (arrows in F). Co‐labeling with pS6 showed consistent co‐localization with SPI1/PU.1 in malformed cells (H, H_1_). Sections A–C were counterstained with hematoxylin. Scale bars: 50 µm in A, E, H, 5 µm in insert B_1_ (representative for B_2_, C_1_)_,_ 10 µm in C_2_, H_1_; arrows = malformed cells, arrowheads = microglia. D Mann–Whitney U test. Data are expressed relative to expression observed in autopsy controls and displayed as Tukey's box plot; * *p* < 0.05, ** *p* < 0.01. n = 8 autopsy control (for both FCD 2b and TSC control groups) versus n = 10 TSC and n = 8 FCD 2b samples

After identifying SPI1/PU.1 as a key transcriptional regulator in TSC tubers, we validated its expression in TSC tuber tissue and the closely related mTORopathy FCD 2b. In autoptic control tissue, SPI1/PU.1 expression was restricted exclusively to microglial nuclei (Figure 2A,A1 arrowheads). In contrast, dysmorphic neurons and balloon/giant cells (referred to as malformed cells) in FCD 2b and TSC displayed strong expression of SPI1/PU.1 (Figure 2B,B1,C). These findings were validated and confirmed using another SPI1/PU.1 antibody (Figure S1D,E, arrows). As for microglia, nuclear SPI1/PU.1 expression appeared stronger and the overall number of microglia was higher in FCD 2b and TSC (Figure 2B,C, arrowheads). Of note, SPI1/PU.1 expression could also be found localized in the nucleus of malformed cells and occasionally dysmorphic neurons, confirming its translocation to the nucleus (Figure 2B2,C1,C2,F). We found no SPI1/PU.1 expression in CD3‐positive T‐lymphocytes (not shown). To further validate our findings from the *in silico* analysis we investigated a separate cohort of TSC cortical tubers and surgically resected FCD 2b tissue and confirmed higher *SPI1* expression compared to autoptic control tissue (Figure 2D). Next, to unravel the cell‐specific localization of SPI1/PU.1, we performed double labeling with cell markers for activated microglia (HLA‐DR), astrocytes (GFAP), neurons (NeuN), and mTOR activation (pS6). We found SPI1/PU.1 expression exclusively in microglial nuclei and malformed cells that occasionally co‐localized with GFAP in TSC and FCD 2b tissue (Figure 2E–G). Additionally, SPI1/PU.1 in malformed cells co‐localized with pS6, indicating mTOR hyperactivation; however, the signal strength of SPI1/PU.1 was heterogeneous in the population of malformed cells (Figure 2H).

Since we observed a strong positive correlation between *SPI1* and *IRF8* (*ρ* = 0.87, *p*‐value = 1.54 × 10^−7^) (Figure S2A) and as it has previously been described that SPI1/PU.1 works in unison with Irf8 to regulate the development and activation of microglia (41, 42), we were interested if the same TF axis of SPI1/PU.1 and Irf8 was active in the malformed cells of TSC and FCD 2b. First, we assessed the expression of *IRF8* in a cohort of surgically resected FCD 2b tissue and showed that similarly to *SPI1*, the expression of *IRF8* was also higher compared to autoptic control tissue (Figure S2B). Next, to assess localization of the two TFs double labeling of SPI1/PU.1 and IRF8 was performed in TSC and FCD 2b tissue. IRF8 expression was detected in the nucleus of microglial (arrowheads) and giant and balloon cells (arrows) (Figure S2C,D). In malformed cells, IRF8 expression co‐localized with SPI1/PU.1 (Figure S2C_1_,D_1_,D_2_). Collectively, these results suggest that the TFs SPI1/PU.1and IRF8 are co‐expressed in malformed cells.

### 3.3 SPI1/PU.1 expression in malformed cells is present early in development and precedes seizure development

**FIGURE 3:**
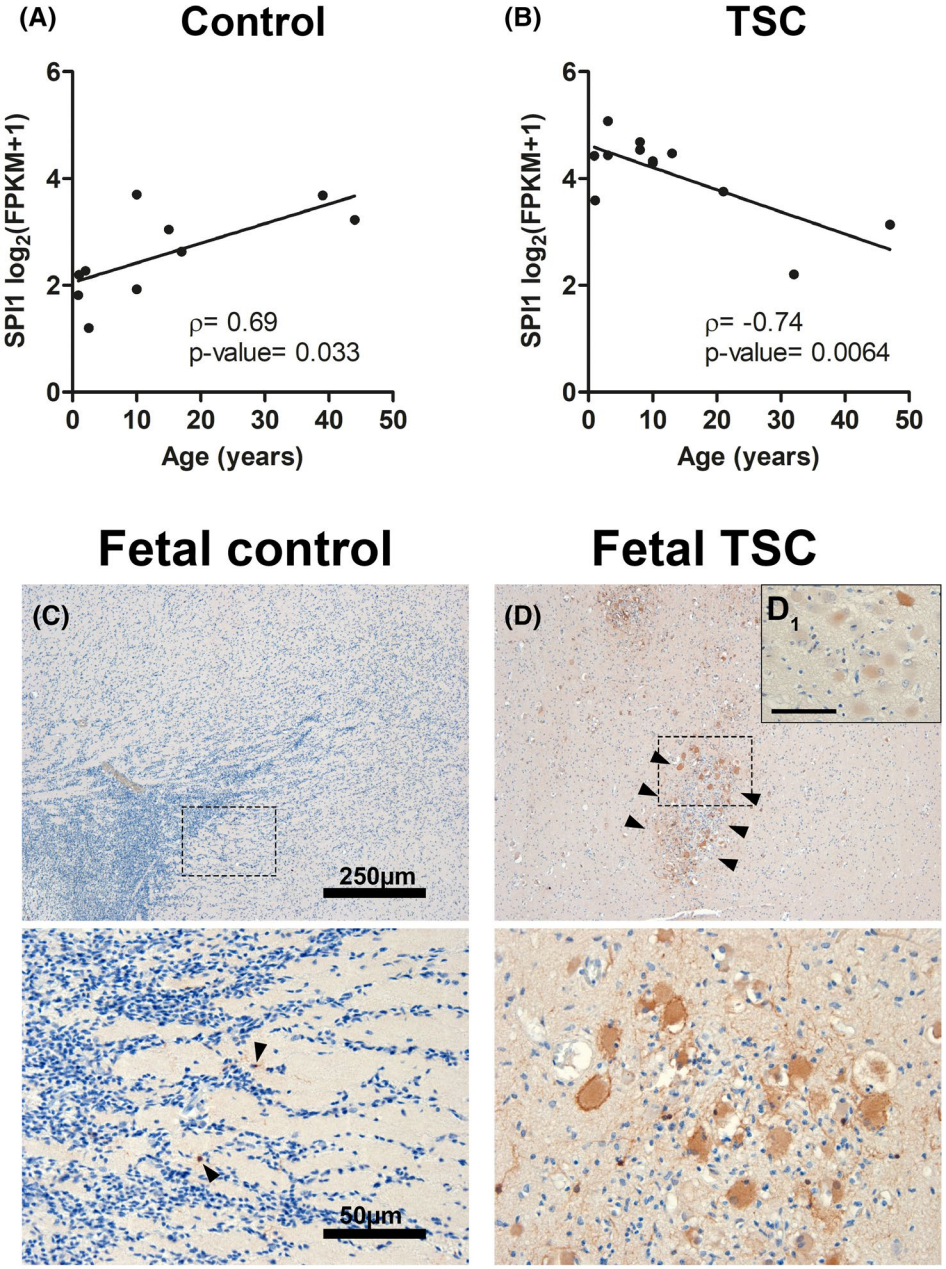
SPI1/PU.1 expression in malformed cells occurs early in TSC development. *SPI1* expression displayed a significant positive correlation with age in control samples (A). In contrast, TSC tuber tissue displayed a negative correlation with age samples (Pearson's correlation) (B). Fetal control cortex displayed sparse SPI1/PU.1 expression solely in few microglia (arrowheads in magnified lower panel) (C). Fetal TSC cases revealed strong expression of SPI1/PU.1 in malformed cells that could be found in subcortical clusters (arrowheads, magnified in lower panel) (D). SPI1/PU.1 expression was found to be variable between malformed cells (D_1_). Sections C and D were counterstained with hematoxylin. Scale bars: 250 µm in upper panel and 50 µm in C (representative for D), 25 µm in D_1_; A, B Spearman's rank correlation test (displayed with Spearman correlation coefficient ρ and exact *p*‐value)

To assess the effect of age on *SPI1* expression, we correlated age with *SPI1* expression as assayed by RNA Seq. While *SPI1* was positively correlated (*ρ* = 0.69, *p*‐value = 0.033) with the age of the control cases, it was negatively correlated with the age of patients with TSC cortical tubers (*ρ* = −0.74, *p*‐value = 0.0064) (Figure 3A,B). This suggests that upregulation of *SPI1* was most prominent in the younger TSC samples. As such, we investigated if SPI1/PU.1 dysregulation represents an early pathogenic mechanism by analyzing fetal TSC tissue obtained from autopsy. We found SPI1/PU.1 expression only in very few, sparsely distributed cells with microglial morphology in fetal control tissue (Figure 3C, arrowheads). In comparison, fetal TSC tissue displayed SPI1/PU.1 expression in clusters of malformed cells that could be found in the white matter or adjacent to the developing cortex (Figure 3D). As in postnatal TSC tissue, malformed cells displayed heterogeneous expression patterns of SPI1/PU.1 (Figure 3D1).

### 3.4 Spi1 RNA but not protein expression precedes the development of seizures in *Tsc1* ^GFAP−/−^ mice

**FIGURE 4:**
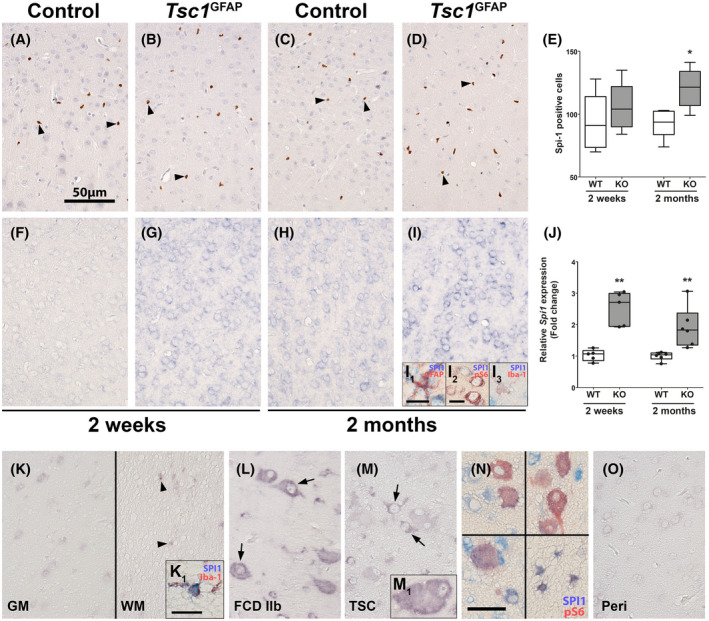
Spi1/Pu.1 RNA expression in the cortex is elevated in *Tsc1* ^GFAP−/−^ mice, while protein expression increases with microglial cell number. Antibody staining against Spi1/Pu.1‐expressing cells in the cortex of *Tsc1* ^GFAP−/−^ mice was not different in 2‐week‐old animals between WT and KO and restricted to microglia (A, B). In contrast, the number of Spi1/Pu.1‐expressing cells was higher in KO animals at 2 months (C, D). Quantification of Spi1/Pu.1‐expressing cells in the cortex confirmed higher Spi1/Pu.1‐positive cell count in 2‐month‐old *Tsc1* ^GFAP−/−^ animals (E). *In situ* hybridization against *Spi1* in the cortex of *Tsc1* ^GFAP−/−^ mice showed higher expression of *Spi1* at 2 weeks of age in all cell types before seizure development compared to age‐matched animals, where expression was absent (F, G). In 2‐month‐old animals *Spi1* expression appeared higher in control animals compared to 2‐week‐old mice (H). In 2‐month‐old animals that suffered from spontaneous recurrent seizures, very strong Spi1 expression could be detected in astrocytes, microglia, and pS6‐positive neurons (I, I_1‐3_). Quantification of total Spi1 RNA confirmed higher Spi1 RNA expression in *Tsc1* ^GFAP−/−^ animals compared to age‐matched littermates (J). *In situ* hybridization in human autopsy tissue revealed very weak expression in cortical neurons, and mostly cells with microglial morphology, as confirmed by co‐labeling with Iba‐1 (K, K_1_). SPI1 in FCD 2b lesions and TSC tubers could be found in malformed cells (L, M, M_1_). Moreover, *SPI1* co‐localized with pS6 expression in malformed cells, but also cells with astrocyte morphology (N). Peri‐lesional tissue displayed expression similar to autopsy control tissue (O). Sections A–D were counterstained with hematoxylin. Scale bars: 50 µm in A (representative for B‐D, F‐I, K‐M, O)_,_ 25 µm in N, 15 µm in I_2_, 10 µm in I_1_ (representative for I_3_) and K_1_ (representative for M_1_) arrowheads in A = microglia, arrowheads in K = glia, arrows in L, M = malformed cells. E, J Mann–Whitney U test. Data are expressed relative to expression observed in age‐matched wild‐type controls and displayed as Tukey's box plots; * *p* < 0.05, ** *p* < 0.01. n = 5 wild‐type animals versus n = 6 *Tsc1* ^GFAP−/−^ mice

To further investigate the role of SPI1/PU.1 in the transcriptional network of TSC, we employed the Tsc1^GFAP−/−^ mouse model which presents with delayed seizure onset, resulting from conditional Tsc1 loss‐of‐function in GFAP‐expressing cells. The delay in seizure onset indicates that other factors additional to the principal mutation and resulting cell autonomous mTOR hyperactivation cause epilepsy, such as alterations in the transcriptional profile over time. Though this model does not recapitulate all the histological features of human TSC, the conditional KO of the *Tsc1* gene in GFAP‐expressing cells during brain development leads to consistent development of spontaneous recurrent seizures. Moreover, seizure development occurs with a delay of approximately 4–6 weeks after birth, so this model allows analysis of potentially epileptogenic processes caused by mTOR activation in the time period before seizure‐onset. Spi1/Pu.1 expression was exclusively detected in microglia in the cortex of control and *Tsc1* ^GFAP−/−^ animals (Figure 4A–D). Overall, the number of Spi1/Pu.1‐expressing cells between WT and KO was similar in 2‐week‐old animals, while 2‐month‐old *Tsc1* ^GFAP−/−^ animals displayed higher numbers of Spi1/Pu.1‐positive microglia (Figure 4E). Employing *in situ* hybridization, *Spi1* expression could not be detected in 2‐week‐old WT animals, but in neurons and glia in 2‐month‐old WT animals (Figure 4F,H). Relative to WT animals, *Tsc1* ^GFAP−/−^ animals displayed higher expression of *Spi1* in neurons and glia (Figure 4G,I). Double labeling confirmed co‐localization of *Spi1* with GFAP, pS6, and Iba‐1 (Figure 4I1,I2,I3). Quantification of total RNA expression in *Tsc1* ^GFAP−/−^ mice revealed higher expression of *Spi1* in the cortex of 2‐week‐old mice prior to development of seizures which persisted to the age of 2 months when spontaneous recurrent seizures occurred (Figure 4J). In human autopsy control tissue, *SPI1* could be detected faintly in gray matter neurons, as well as cells with (micro‐)glial morphology in the white matter using *in situ* hybridization (Figure 4K,K1). In FCD 2b and TSC tissue, we found higher *SPI1* expression in dysmorphic neurons and some balloon/giant cells (Figure 4L,M,M1). Additionally, SPI1 was detected in pS6‐positive balloon/giant cells and white matter astrocytes (Figure 4N). In contrast, perilesional tissue displayed faint neuronal expression similar to control (Figure 4O). These findings support that SPI1 RNA expression is elevated in malformed cells and glia TSC. Moreover, *SPI1* expression appears to be increased consistently in *Tsc1* KO animals, while protein expression is limited to microglial nuclei.

### 3.5 Oxidative stress is evident in TSC tissue and represents a driver for SPI1 transcription *in vitro* in various cells

**FIGURE 5:**
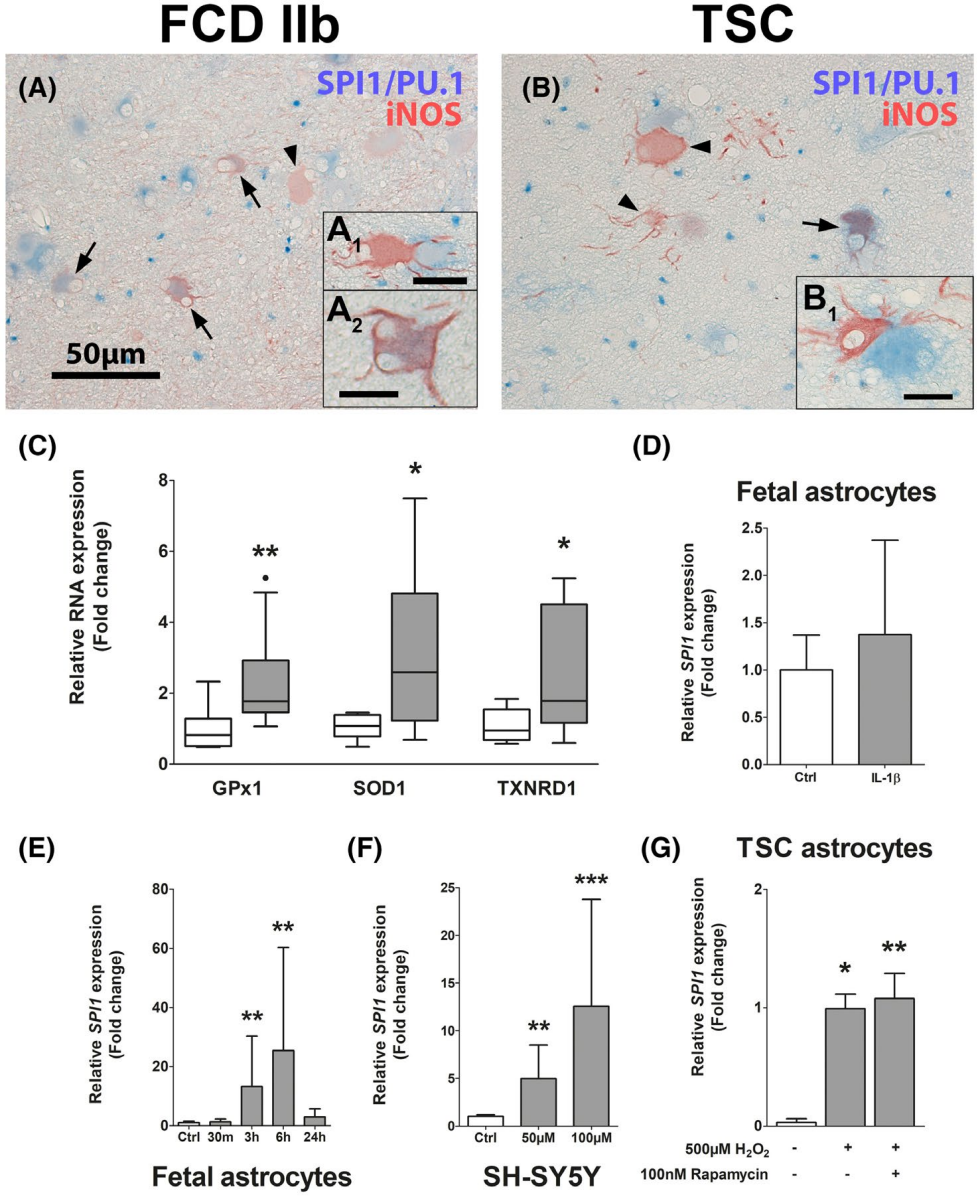
Oxidative stress is present in malformed cells in TSC cortical tubers and stimulates *SPI1* expression *in vitro*. Expression of iNOS could be detected exclusively in malformed cells in FCD 2b and TSC tissue. Co‐localization of SPI/PU.1 and iNOS was found in some malformed cells (arrows in A, B and A_2_). Moreover, iNOS expression was often found in cells that were in close proximity to SPI1/PU.1‐expressing cells (A_1_, B_1_) that wrapped around each other (A, B). Analysis of the OS markers *GPx1*, *SOD1*, and *TXNRD1* showed higher expression in TSC tissue compared to control (C). Human fetal astrocytes did not display higher *SPI1* expression when exposed to IL‐1β, but H_2_O_2_ in a time‐dependent manner (D, E). SH‐SY5Y neuroblastoma cells exposed to H_2_O_2_ displayed *SPI1* induction, similar to astrocytes (F). *In vitro*, TSC‐derived astrocytes displayed higher expression of *SPI1* when exposed to H_2_O_2_, which was independent of mTOR inhibition via rapamycin (G). Scale bars: 50 µm in A_,_ 25 µm in A_1_, 20 µm in B_1_, 10 µm in A_2_, arrowheads = iNOS single positive cells, arrows = co‐localized cells. (C and D) Mann–Whitney U test. (E–G) Kruskal–Wallis test with Dunn's *post hoc*. (C) Data are expressed relative to expression in autopsy control and displayed as Tukey's box plot. (D–G) Data are expressed as bar graphs with SD; *p < 0.05, **p < 0.01, ***p < 0.001. (C) n = 8 autopsy control versus n = 10 TSC samples. (D–G) n = 3 independent cultures in duplicates

After we identified SPI1/PU.1 expression to be upregulated in malformed cells and its involvement early in TSC development, we investigated which pathogenic stimuli might drive the expression of SPI1/PU.1. While brain inflammation is a well characterized phenomenon in cortical tubers of TSC patients, we also found expression of the pro‐oxidant enzyme iNOS in malformed cells and activation of anti‐oxidant gene expression in whole TSC tissue, indicative of OS (Figure 5A–C). Here, we detected some cells displaying co‐expression of SPI/PU.1 and iNOS, but also iNOS‐positive cells surrounded by SPI/PU.1‐expressing cells (Figure 5A1,A2,B1). These cells did not present with typical dysmorphic neuron or balloon/giant cell morphology, but rather presented with dysplastic, process‐rich, astrocytic morphology. We used human fetal astrocytes to further characterize *SPI1* expression in response to TSC‐relevant stimuli. Since we found a strong correlation between *IL*‐*1β* and *SPI1* (*ρ* = 0.817) in the sequencing dataset we investigated if IL‐1β can induce SPI1/PU.1. Therefore, we stimulated fetal astrocytes with IL‐1β to study if pro‐inflammatory stimuli regulate SPI1; however, we could not detect any differences in SPI1 expression (Figure 5D). Moreover, stimulation of fetal astrocytes with H_2_O_2_ led to a time‐dependent upregulation of *SPI1*, similar to TSC‐derived astrocytes (Figure 5E). Surprisingly, *SPI1* expression could even be induced in the neuroblastoma cell line SH‐SY5Y upon OS in a concentration‐dependent manner (Figure 5F). Furthermore, to test if mTOR hyperactivation *per se* drives *SPI1* expression, we analyzed *SPI1* expression in astrocytes derived from TSC tubers. Additionally, we stimulated TSC astrocytes with H_2_O_2_ only or in combination with the mTOR inhibitor rapamycin to untangle if OS and/or mTOR hyperactivation are involved in *SPI1* expression. Interestingly, we found a strong induction of *SPI1* by H_2_O_2_ in TSC astrocytes, which could not be blocked by rapamycin treatment (Figure 5G). Importantly, *SPI1* in one of three unstimulated TSC‐astrocyte cultures was below detection level. Collectively, these results indicate that OS strongly induces *SPI1* RNA expression in a multitude of cell types, and that this stimulation on the RNA level is independent of cell‐autonomous mTOR hyperactivity in TSC‐derived cells.

## 4 DISCUSSION

In this study, we investigated the transcriptional regulation of the TSC tuber environment and identified the transcription factor SPI1/PU.1 as pathogenic transcriptional regulator in TSC and could confirm its dysregulation in the related pathology FCD 2b. We found strong correlation with a variety of pro‐inflammatory mediators, making SPI1/PU.1 a candidate driver of brain inflammation. SPI1/PU.1 expression was concentrated in malformed cells in lesions and its upregulation could be seen during fetal development. Moreover, we found SPI1/PU.1 expression in dysmorphic cells with mTOR activation and in glia in TSC tubers, FCD 2b lesions, and prenatal TSC brain tissue, which concur with higher RNA expression in *Tsc1* ^GFAP−/−^ mice. Moreover, we found that OS potently drives the transcriptional activation of *SPI1* in a variety of cells *in vitro*, including TSC‐derived astrocytes. While we identified elevated *SPI1* RNA expression, likely promoted via OS, SPI1/PU.1 protein translation appears to critically rely on translation defects specific to mTOR activation in malformed cells TSC and FCD 2b lesions.

Identification of TFs that are major regulators of the underlying pathogenesis of TSC could aid in the development of new therapeutic tools that could modify the disease course in TSC and other mTORopathies. Here, we employed various unbiased *in silico* approaches across previously produced RNA Seq datasets to identify the transcriptional regulators that potentially influence the differential expressions pattern seen in TSC. We extracted four upregulated TFs from the data: SPI1/PU.1, IRF8, GBX2, and IKZF1. After an enrichment analysis of the upregulated genes for TF targets, we did not detect significant enrichment of upregulated genes for GBX2 and IKZF1, thus we did not focus further on these TFs. However, since little is known about the function of these TFs, the data available on the reference repositories employed for gene enrichment analysis is limited, influencing the outcome of this analysis. Nevertheless, these TFs might serve important modulatory functions in TSC tubers. Moreover, we aimed to identify novel drivers of gene expression in TSC and not repressors. For instance, GBX2 was shown to have important roles as a transcriptional repressor in telencephalic development in zebrafish (43, 44) and specifically development of forebrain GABAergic neurons in mice (45). Moreover, IKZF1 is a transcriptional regulator of erythropoiesis (46) and, in the brain, was implicated in transcriptional alterations in neurodegeneration, neuronal differentiation, and functions of the endocrine system (47, 48, 49, 50). Interestingly, syndromes caused by deletions of IKZF1 present with intellectual disability (51). Taken together, upregulation of GBX2 and IKZF1 could still reflect important changes in the aberrant brain development and syndromes of TSC by mild modulation of neural development genes below the cut‐off *p*‐value or by acting as repressors. Furthermore, it is important to consider the contribution of the altered cellular composition of the tuber, as GBX2 upregulation could also reflect lymphocyte infiltration, a feature commonly observed in TSC tubers (52, 53). While other TFs previously implicated in TSC, such as tp53 and NF‐κB, were not upregulated, we still analyzed gene enrichment for these TFs (21, 24, 54, 55). We could retrieve data only for RelA for which a significant enrichment value was calculated. Since this transcription factor is involved in the activation of a number of pro‐inflammatory genes, its enrichment in TSC tuber was anticipated from previous studies (12, 55). In *Tsc2* ^−/−^ zebrafish, we detected upregulation of *Spi1b*, *Ikzf1*, *tp53*, and *Rel*, as well as *RelB* downregulation, while gene enrichment was computed for *Spi1b*, *Irf8*, and *RelA*. Because we detected upregulation and enrichment of upregulated genes for *SPI1* and its homolog in both human and zebrafish data, we focused our attention on SPI1 as transcriptional driver. We did not focus on NF‐κB TFs since these were already previously characterized in TSC (21, 55).

SPI1/PU.1 is a transcriptional activator, known to regulate the expression of hundreds of genes by binding to a purine‐rich sequence, known as PU‐box that is found adjacent to the promoter region of its target genes (56, 57, 58). More specifically, SPI1/PU.1 is integral for the maturation of microglia, lymphocytes, and the majority of myeloid cell lines. As such, SPI1/PU.1 is critical to the maturation and regulation of the immune system and brain inflammation, two processes that are pathogenic hallmarks of TSC (59, 60, 61). Furthermore, SPI1/PU.1 has been instigated in leukemia, Alzheimer's disease and, notably in the context of epileptogenic pathologies, in the chronic transcriptional changes that result from experimental traumatic brain injury (TBI) in rats (62, 63, 64). In our data set, SPI1/PU.1 enriched for genes involved in Toll‐like receptor (TLR) signaling, complement activation, and cytokine signaling, all of which support the pro‐inflammatory environment in TSC and SPI1/PU.1 as potential activator of these processes. Importantly, these processes were shown to be activated in malformed cells in TSC tubers previously (52, 65, 66). Since innate and adaptive immune cells play a role in these inflammatory processes, we wondered if peripheral immune cells and microglia, malformed cells with cell‐autonomous mTOR activation or all cell types contribute to the inflammatory environment in TSC tubers.

Since SPI1/PU.1 in microglia is essential for viability and phenotypic differentiation, its expression in microglia was expected (41, 67). While more activated microglia can be found in tubers, we also localized SPI1/PU.1 expression to malformed cells in TSC and FCD 2b, respectively. Moreover, we also identified IRF8 and its target genes to be upregulated in cortical tubers, a factor that is essential for microglia development and activation (41, 42). This suggests that increased microglial numbers and their activation contribute to the higher expression of SPI1/PU.1 and its target genes, a feature previously demonstrated in TSC tubers (52, 68). However, we also detected the expression of these factors in malformed cells. It is noteworthy, that despite SPI1/PU.1 expression in microglia usually being localized strictly to the nucleus, in malformed cells we detected frequently cytoplasmic expression. One reason could be a disturbed redox balance caused by OS, preventing regulated nuclear translocation of SPI1/PU.1 in malformed cells as shown by a previous study (69). Nevertheless, we still detected malformed cells with nuclear expression, thus potentially active SPI1/PU.1.

Interestingly, SPI1/PU.1 expression is already elevated in fetal TSC brain tissue in malformed cells, before terminal differentiation of microglial cells at approximately gestational week (GW) 35 (70, 71, 72, 73). The fetal TSC cases included in our study were GW 27 and GW 32 (GW32 in the representative picture), respectively. This indicates that malformed cells already display SPI1/PU.1 expression before brain‐wide infiltration, differentiation, and potentially activation of microglia, enabling pro‐inflammatory transcriptional programs to be activated in malformed cells already during development. In support of this, conditional KO of *Tsc1* in *Tsc1* ^GFAP−/−^ also induced an early transcription of *Spi1*. Interestingly, we found wide‐spread expression of *Spi1* in the cortex of *Tsc1* ^GFAP−/−^ mice, in astrocytes, neurons, and microglia. In contrast, Spi1/Pu.1 protein expression could only be localized to microglial nuclei.

SPI1/PU.1 is a TF solely restricted to microglia and infiltrating immune cells in the healthy brain and PU.1 is frequently used as microglia/monocyte marker in the brain. Thus, we anticipated that mTOR hyperactivation in malformed cells would be the aberrant driving force for SPI1/PU.1 expression in malformed cells. Remarkably, we found OS to strongly induce *SPI1* expression in a time‐ and concentration‐dependent manner in astrocytes and the SH‐SY5Y neuroblastoma cell line. Most importantly, inhibition with rapamycin in TSC‐derived astrocytes had no effect on increased *SPI1* expression, suggesting mTOR independent activation of *SPI1* RNA expression. While we cannot rule out secondary inflammatory signaling in response to OS (74), direct receptor‐mediated pro‐inflammatory stimulation with IL‐1β, that normally induces a robust inflammatory response in fetal astrocytes (40, 75), did not alter SPI1/PU.1 expression. Moreover, we detected upregulation of antioxidant genes, an indicator of elevated OS, in tuber tissue in agreement with previous studies (12, 17). Importantly, expression of OS response genes characterized in previous studies coincides with *SPI1* expression in fetal TSC and in the brain of *Tsc1* ^GFAP−/−^ mice (17). Hence, we conclude that OS is a driver of SPI1/PU.1 RNA expression, which is supported by previous studies showing that TFs induced by OS can stimulate *SPI1* expression (76). Moreover, OS is a potent activation signal for a multitude of TFs and could lead to their translocation and transcriptional activation of *SPI1* transcription (77). Lastly, a previous report demonstrated a correlation between hypomethylation of IL‐1β promotor regions and its higher expression in TSC tubers (20). Hence, alterations in the methylation of the SPI1 promotor might be an additional mechanism that could promote aberrant SPI1 expression in malformed cells.

While we detected robust RNA expression across *in vitro* and *in vivo* models, we did not detect Spi1/Pu.1 protein expression in cells other than microglia in *Tsc1* ^GFAP−/−^ mice. However, while *Spi1* was elevated already at 2 weeks of age, the number of microglia was not increased, only after the development of seizures at 2 months, in agreement with previous reports about this model (78). This suggests that OS caused by mTOR activation stimulates *Spi1* mRNA expression in a variety of cells, but other posttranscriptional regulatory mechanisms inhibit Spi1/Pu.1 translation. While initial *SPI1* RNA expression appears to be driven by OS, translational dysregulation of *SPI1* mRNA might require a trigger present in cells with aberrant cell‐autonomous mTOR activation, such as malformed cells. The lack of protein translation suggests that Spi1/Pu.1 in *Tsc1* ^GFAP−/−^ mice does not play the same pro‐inflammatory role as in TSC tubers from patients. However, this model and the zebrafish model confirm that an environment of mTOR hyperactivation promotes OS‐mediated *Spi1* expression. Moreover, microglia‐specific upregulation of PU.1 in response to OS could certainly exert pro‐inflammatory effects secondary to mTOR hyperactivation.

We hypothesize that mTOR activation in malformed cells predisposes specifically this cell population to *SPI1* translation caused by promotion of protein synthesis and suppression of protein catabolism by mTOR complex 1 (79). Since replication of malformed cells and tubers in TSC models is still lacking, it is currently impossible to model this particular aspect. Interestingly, the precursor of malformed cells is hypothesized to be a human‐specific radial glia cell population, that could only be replicated in human‐derived organoid models (80). To ultimately verify our hypothesis, ChIP‐Seq experiments should be performed on malformed cells of tuber tissue or derived from TSC‐derived organoids to confirm binding of SPI1/PU.1 to putative transcriptional targets. While it was shown that it is possible to isolate and culture malformed cells *in vitro* (81), only few cells can be isolated and large‐scale ChIP‐Seq experiments are, therefore, not feasible. Moreover, replicating malformed cells and their characteristic features *in vitro* remains difficult to date. While we analyzed *SPI1* expression in TSC‐derived astrocytes, these cells do not recapitulate all the features of dysmorphic neurons and giant/balloon cells, the cells displaying the strongest SPI1/PU.1 expression. Moreover, it remains questionable if the tuber microenvironment is properly replicated *in vitro* and we assume this factor also plays an essential part in mediating aberrant protein translation in malformed cells.

While we acknowledge the contribution of microglia and other leukocytes to SPI1/PU.1 upregulation in TSC tubers, we conclude that SPI1/PU.1 in malformed cells with intrinsic mTOR activation drives the execution of immunogenic transcriptional programs normally only present in microglia, promoting the expression of pro‐inflammatory factors such as complement or cytokines. Thus, by specifically targeting this transcription factor it could be possible to dampen inflammation mediated by these cells in TSC and other mTORopathies like FCD 2b.

## CONFLICT OF INTEREST

The authors declare that they have no conflict of interest.

## AUTHOR CONTRIBUTIONS

Till S. Zimmer, Anatoly Korotkov, Susan Zwakenberg, Fried J. T. Zwartkruis, and James D. Mills performed the experiments, data collection, and analysis. Floor E. Jansen, Angelika Mühlebner, Nicholas R. Rensing, Michael Wong, Erwin A. van Vliet, James D. Mills, and Eleonora Aronica helped with the selection and collection of brain tissues and clinical data. James D. Mills, Erwin A. van Vliet, and EA conceived the study and participated in its design and coordination. Till S. Zimmer, James D. Mills, Erwin A. van Vliet, and Eleonora Aronica drafted and prepared the manuscript. All authors read, revised, and approved the final manuscript.

## CONSENT FOR PUBLICATION

All authors read and agreed with the contents of this paper.

## ETHICS APPROVAL AND CONSENT TO PARTICIPATE

All procedures performed in studies involving human participants were in accordance with the Amsterdam UMC Research Code provided by the Medical Ethics Committee and with the 1964 Helsinki declaration and its later amendments or comparable ethical standards. All procedures performed in studies involving animals were in accordance with the ethical standards of the Washington University Animal Welfare committee.

## Supporting information

FIGURE S1. Expression of transcriptional regulators in TSC cortical tubers and *tsc*2^−/−^ zebrafish and SPI1/PU.1 validation. Expression of TP53 and NF‐κB subunits was not different between TSC tuber tissue compared to autopsy control (A). In zebrafish *tsc2* ^−/−^ mutants, the homolog *spi1b* and *ikzf1* were higher compared to control zebrafish, while *irf8* and *gbx2* were not different (B). Additionally, *tp53* and *rel* expression were higher in mutant zebrafish while *relb* expression was lower compared to the control (C). Localization to microglial nuclei and stronger staining in malformed cells was detected using anti‐PU.1 from Thermo Fisher Scientific (D, E). Sections D and E were counterstained with hematoxylin. Scale bars: 50 µm in D (representative for E)_,_ 20 µm in inserts D_1_ and E_1_; arrowheads = microglia, arrows = malformed cells. A Data are expressed relative to expression in autopsy control and displayed as Tukey's box plot. RNA Seq n = 10 autopsy control cortex samples (clear) versus n = 12 TSC cortical tubers (gray). (B and C) Data are expressed as bar graphs with SD. n = 4 WT zebrafish (clear) versus n = 3 *tsc2* ^GFAP−/−^ zebrafish (gray). *p < 0.05, modified *t* test

FIGURE S2. *IRF8* is increased in FCD 2b and TSC, and co‐localizes with *SPI1* in dysmorphic cells. *SPI1* and *IRF8* expression from RNA sequencing data display strong positive correlation (A). Total *IRF8* expression was higher in FCD 2b tissue and a separate cohort of TSC (B). IRF8 expression was detected in the nucleus of microglia (arrowheads) and malformed cells (arrows) in FCD 2b and TSC tissue (C, D). IRF8 expression in giant and balloon cells was co‐localized with cytoplasmic SPI/PU.1 expression (C_1_, D_1,2_). Sections C and D were counterstained with hematoxylin. Scale bars: 50 µm in C_,_ 10 µm in C_1_ (representative for inserts B_1,2_), arrowheads = microglia, arrows = malformed cells. (A) Spearman's rank correlation test (displayed with Spearman correlation coefficient *ρ* and exact *p*‐value). (B) Data are expressed relative to expression in autopsy control and displayed as Tukey's box plot. Mann–Whitney U test. *p < 0.05. (A) n = 10 autopsy control cortex samples versus n = 12 TSC cortical tubers. (B) n = 8 autopsy control (for both FCD 2b and TSC control groups) versus n = 10 TSC and n = 8 FCD 2b samples

Table S1‐S4
**TABLE S1** Clinical information of autopsy control tissue
**TABLE S2** Clinical information of FCD IIb and TSC tissue
**TABLE S3** Oligonucleotide sequence of SPI1 probe
**TABLE S4** Primer sequences used for quantitative real‐time PCR

Supplementary Material

## Data Availability

The original RNA sequencing data utilized for the identification of the transcription factors can be found in the primary publication (https://doi.org/10.1038/s41598‐017‐06145‐8). The datasets generated and/or analyzed during the current study are available in the European Genome‐phenome Archive, using accession codes for primary (EGAS00001002485) and secondary (GSE67835) RNA sequencing data.
