# Supplementary figures and images for: Upregulation of the pathogenic transcription factor SPI1/PU.1 in tuberous sclerosis complex and focal cortical dysplasia by oxidative stress
Source: Brain Pathol. 2021 Mar 30;31(5):e12949. doi: 10.1111/bpa.12949 (PMC8412124; doi:10.1111/bpa.12949)

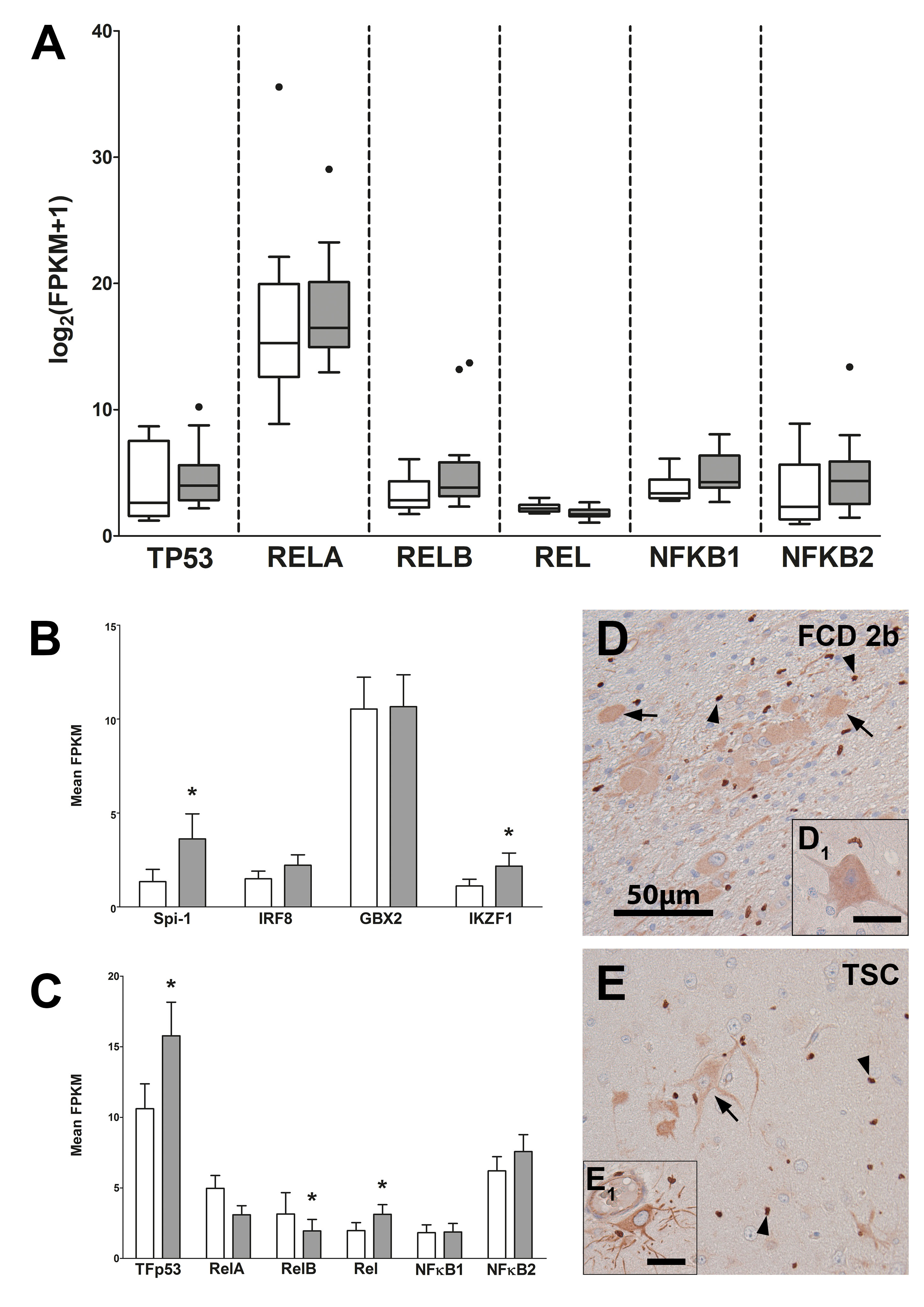

Supplement: Supplementary file 1 — FIGURE S1. Expression of transcriptional regulators in TSC cortical tubers and tsc2−/− zebrafish and SPI1/PU.1 validation. Expression of TP53 and NF‐κB subunits was not different between TSC tuber tissue compared to autopsy control (A). In zebrafish tsc2 −/− mutants, the homolog spi1b and ikzf1 were higher compared to control zebrafish, while irf8 and gbx2 were not different (B). Additionally, tp53 and rel expression were higher in mutant zebrafish while relb expression was lower compared to the control (C). Localization to microglial nuclei and stronger staining in malformed cells was detected using anti‐PU.1 from Thermo Fisher Scientific (D, E). Sections D and E were counterstained with hematoxylin. Scale bars: 50 µm in D (representative for E), 20 µm in inserts D1 and E1; arrowheads = microglia, arrows = malformed cells. A Data are expressed relative to expression in autopsy control and displayed as Tukey's box plot. RNA Seq n = 10 autopsy control cortex samples (clear) versus n = 12 TSC cortical tubers (gray). (B and C) Data are expressed as bar graphs with SD. n = 4 WT zebrafish (clear) versus n = 3 tsc2 GFAP−/− zebrafish (gray). *p < 0.05, modified t test [file BPA-31-e12949-s001.jpg]

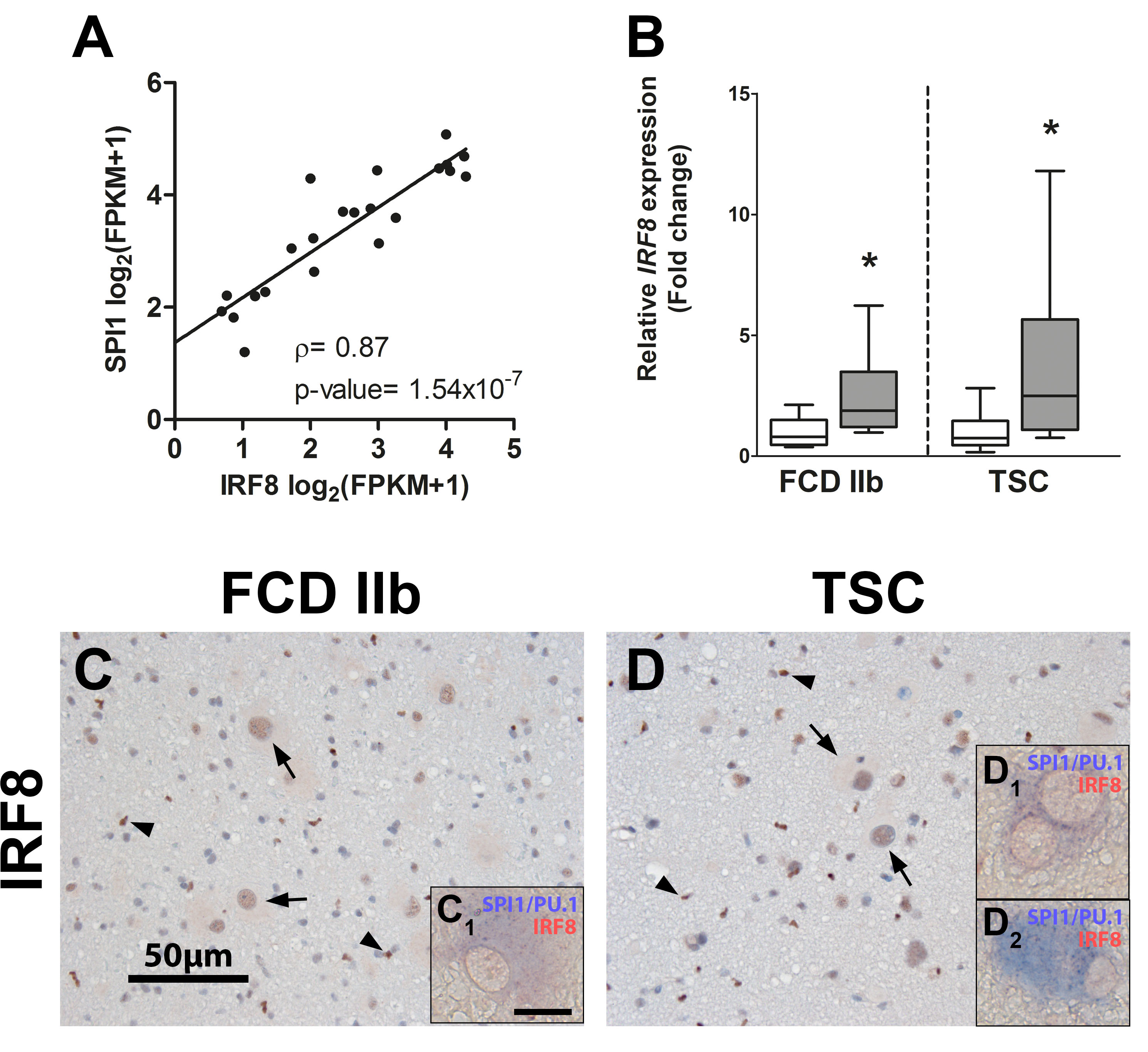

Supplement: Supplementary file 2 — FIGURE S2. IRF8 is increased in FCD 2b and TSC, and co‐localizes with SPI1 in dysmorphic cells. SPI1 and IRF8 expression from RNA sequencing data display strong positive correlation (A). Total IRF8 expression was higher in FCD 2b tissue and a separate cohort of TSC (B). IRF8 expression was detected in the nucleus of microglia (arrowheads) and malformed cells (arrows) in FCD 2b and TSC tissue (C, D). IRF8 expression in giant and balloon cells was co‐localized with cytoplasmic SPI/PU.1 expression (C1, D1,2). Sections C and D were counterstained with hematoxylin. Scale bars: 50 µm in C, 10 µm in C1 (representative for inserts B1,2), arrowheads = microglia, arrows = malformed cells. (A) Spearman's rank correlation test (displayed with Spearman correlation coefficient ρ and exact p‐value). (B) Data are expressed relative to expression in autopsy control and displayed as Tukey's box plot. Mann–Whitney U test. *p < 0.05. (A) n = 10 autopsy control cortex samples versus n = 12 TSC cortical tubers. (B) n = 8 autopsy control (for both FCD 2b and TSC control groups) versus n = 10 TSC and n = 8 FCD 2b samples [file BPA-31-e12949-s003.jpg]
