# Supplementary material for: Upregulation of the pathogenic transcription factor SPI1/PU.1 in tuberous sclerosis complex and focal cortical dysplasia by oxidative stress
Source: Brain Pathol. 2021 Mar 30;31(5):e12949. doi: 10.1111/bpa.12949 (PMC8412124; doi:10.1111/bpa.12949)
Supplement: Supplementary file 3 — Table S1‐S4 TABLE S1 Clinical information of autopsy control tissue TABLE S2 Clinical information of FCD IIb and TSC tissue TABLE S3 Oligonucleotide sequence of SPI1 probe TABLE S4 Primer sequences used for quantitative real‐time PCR [file BPA-31-e12949-s002.docx]

**UPRegulation of the pathogenic transcription factor SPI1/PU.1 in tuberous sclerosis complex and FOCAL cortical dysplasia by oxidative stress**

**Till S. Zimmer^1^, Anatoly Korotkov^1^, Susan Zwakenberg^2^, Floor E. Jansen^3^, Fried J. T. Zwartkruis^2^, Nicholas R. Rensing^5^, Michael Wong^5^, Angelika Mühlebner^1,4^, Erwin A. van Vliet^1,7^, Eleonora Aronica^1,8,*^, James D. Mills^1,9,10,*^**

^1^Amsterdam UMC, University of Amsterdam, Department of (Neuro)Pathology, Amsterdam Neuroscience, Amsterdam, the Netherlands

^2^University Medical Center Utrecht, Center for Molecular Medicine, Molecular Cancer Research, Utrecht, the Netherlands

^3^University Medical Center Utrecht, Department of Pediatric Neurology, Brain Center, Utrecht, the Netherlands

^4^Department of Pathology, University Medical Center Utrecht, Utrecht, the Netherlands

^5^Washington University, Department of Neurology, Saint Louis, MO, United States of America
^7^University of Amsterdam, Swammerdam Institute for Life Sciences, Center for Neuroscience, Amsterdam, the Netherlands

^8^Stichting Epilepsie Instellingen Nederland (SEIN), Heemstede, the Netherlands

^9^Department of Clinical and Experimental Epilepsy, UCL, London, United Kingdom

^10^Chalfont Centre for Epilepsy, Chalfont St Peter, United Kingdom

*shared last authorship

**Suppl. Table 1: Clinical information of autopsy control tissue**

| **Gender** | **Age (years)** | **Brain area** |
| --- | --- | --- |
| m | 4 | T |
| f | 7 m | F |
| m | 3 | F |
| f | 25 | F |
| m | 3.6 m | F |
| f | 10 | F |
| m | 6 w | F |
| f | 2 | T, F |
| f | 1 | T |
| m | 13 | F |
| f | 17 | F |
| f | 7 w | F |
| f | 17 | F |
| m | 20 | F |
| m | 39 | F |
| m | 3 d | F |
| m | 31 | F |
| f | 7 | F |
| m | 22 GW | F |
| f | 21 GW | F |
| m | 31 GW | F |
| f | 0,9 | T |
| f | 2,5 | F |
| m | 15 | F |
| f | 1 | T |
| m | 10 | F |
| f | 39 | F |
| f | 44 | F |

**Suppl. Table 2: Clinical information of FCD IIb and TSC tissue.**

| **Pathology** | **Mutation** | **Gender** | **Age (years)** | **Duration epilepsy** | **Seizure types** | **Brain area** | **Seizures/month** | **AEDs** | **Application** |
| --- | --- | --- | --- | --- | --- | --- | --- | --- | --- |
| TSC | TSC1 | m | 8 m | 5m | FS | F | >50 | VGB | RNA |
| TSC | TSC2 | m | 2 y | 1y | IS | F | - | VGB | RNA |
| TSC | TSC2 | f | 4 y | 3y | FS, GS | F | >50 | VGB | RNA |
| TSC | TSC2 | m | 2 y | 2y | IS | F | - | VGB, CLB | RNA |
| TSC | TSC2 | f | 2 y | 15m | GS | T | >50 | VGB | RNA |
| TSC | TSC1 | f | 6 y | 4y | FS | F | >50 | VGB | RNA |
| TSC | TSC2 | m | 1 y | 1y | IS | F | - | VGB, CLB | RNA |
| TSC | TSC2 | f | 3 y | 2.5y | FS, GS | T | >50 | VGB | RNA |
| TSC | TSC2 | m | 3 y | 2.7y | FS | F | 120 | VGB | RNA |
| TSC | TSC2 | m | 8 m | 8 m | FS | F | 61 | VGB, LEV, CLB | RNA |
| FCD IIb | - | m | 41 | 40y | FA, FB/TC | F | 122 | CBZ, PGB, TPM, CLB | RNA |
| FCD IIb | MTOR | f | 9 | 6 y | FIA, FB/TC | F | 9 | OXC | RNA/IHC |
| FCD IIb | MTOR | m | 14 | 13y | FIA | F | 457 | VPA, PHT, LMT | RNA |
| FCD IIb | MTOR | m | 5 | 2y | FIA, SE | F | 152 | VPA, VGB, PHT | RNA |
| FCD IIb | - | f | 28 | 25y | FA, SE | F | 304 | PHT, CBZ, LMT | RNA |
| FCD IIb | - | m | 14 | 10y | FA | F | 213 | LEV, LMT, CBZ | RNA |
| FCD IIb | - | m | 21 | 14y | FIA, FB/TC | F | 122 | LEV, LCS, OXC, CNP | RNA |
| FCD IIb | MTOR | m | 17 | 14y | FIA | F | 122 | LMT | RNA |
| FCD IIb | - | f | 12 | 10y | FIA | F | 356 | VPA, CLB | IHC, ISH |
| FCD IIb | - | m | 13 | 11.5 | FIA, FB/TC | F | 152 | VPA, CBZ | IHC, ISH |
| TSC | TSC1 | f | 4 y | 10m | FIA | F | 600 | PB, CLB, LEV, VGB, OXC, ZNS | IHC, ISH |
| TSC | - | m | 13 | 13y | FIA, FB/TC | F | 213 | PHT, LEV | IHC, ISH |
| TSC | - | f | 22 | 10y | FIA | F | 609 | CBZ, LEV, CLB, LMT | IHC, ISH |
| Fetal TSC | - | f | 32 GW | - | - | F | - | - | IHC, ISH |
| Fetal TSC | - | m | 23 GW | - | - | F | - | - | IHC, ISH |
| Fetal TSC | - | f | 34 GW | - | - | F | - | - | IHC, ISH |
| TSC | TSC1 | f | 9 | 1y | FIA | F | 122 | OXC, CLB | Cell culture |
| TSC | TSC2 | m | 2 | 2y | FIA | F/P | 304 | LEV, VGB, PB, CLB | Cell culture |
| TSC | TSC2 | m | 2y | 2y | FIA | F | 152 | ZNS | Cell culture |
| TSC | TSC2 | f | 13 | 13 | FA | F | 90 | LTG, CBZ, CLB | RNA seq |
| TSC | TSC1 | m | 8 | 8 | IS | F | 90 | LTG, CLB, TPM | RNA seq |
| TSC | TSC2 | m | 32 | 30 | FA | T | >150 | PHB, MDZ, LEV | RNA seq |
| TSC | TSC1 | f | 21 | 15 | FB/TC | F | 150-300 | VPA | RNA seq |
| TSC | TSC1 | m | 0,9 | 0,8 | FIA | T | 150-300 | CBZ, VPA, VGB | RNA seq |
| TSC | TSC2 | f | 10 | 10 | FA | F | 150-300 | OXC, LTG | RNA seq |
| TSC | TSC2 | m | 47 | 35 | FA, | T | >150 | CBZ, CLB | RNA seq |
| TSC | TSC2 | m | 3 | 3 | IS | F | 120-300 | VGB, LTG, CLB | RNA seq |
| TSC | TSC2 | m | 10 | 8 | FIA | F | 150-300 | VPA, CLB | RNA seq |
| TSC | TSC2 | f | 1 | 0,83 | FIA | F | 240 | VGB | RNA seq |
| TSC | TSC1 | f | 8 | 8 | FIA | F | 180 | VGB, CBZ, VPA | RNA seq |
| TSC | TSC2 | m | 3 | 2 | SPS | T | 180 | VGB, LTG, CLB | RNA seq |

**Suppl. Table 3: Oligonucleotide sequence of SPI1 probe.**

| **Name** | **Nucleotide sequence** |
| --- | --- |
| *SPI1/Spi1* | 5’DIG-IAmUmCITmUmCITmUmGICmGmGITmUmGICmCmCITmUmCIT-3’DIG |

**Suppl. Table 4: Primer sequences used for quantitative real-time PCR.**

| **Species** | **Gene** | **Forward primer** | **Reverse primer** |
| --- | --- | --- | --- |
| human | SPI1 | CTCAGCAGTGATGGGGAGAG | CTGGAGCTCCGTGAAGTTGT |
|  | IRF8 | AGCATGTTCCGGATCCCTTG | CTGGTTCAGCTTTGTCCCCT |
|  | GPx1 | TTCCCGTGCAACCAGTTT | GGACGTACTTGAGGGAATTCTG |
|  | SOD1 | TCATCAATTTCGAGCAGAAGG | GCAGGCCTTCAGTCAGTCC |
|  | TXNRD1 | TTTCGACCCGGTCACACAAA | CAAACACAACGGGCAGATCG |
|  | C1ORF43 | GATTTCCCTGGGTTTCCAGT | ATTCGACTCTCCAGGGTTCA |
|  | EF1α | ATCCACCTTTGGGTCGCTTT | CCGCAACTGTCTGTCTCATATCAC |
| mouse | Spi1 | TCAGAGCTATACCAACGTCCAAT | GTGCGGAGAAATCCCAGTAGT |
|  | Hprt-1 | ATCACATTGTGGCCCTCTG | GTCATGGGAATGGATCTATCACT |
|  | Tbp | GATGGGAATTCCAGGAGTCA | GAGAATCATGGACCAGAACA |
